# Trajectories of Mental Health Status Among Police Recruits in Sweden

**DOI:** 10.3389/fpsyt.2021.753800

**Published:** 2022-02-03

**Authors:** Mikael Emsing, Mojgan Padyab, Mehdi Ghazinour, Anna-Karin Hurtig

**Affiliations:** ^1^Police Education Unit, Umeå University, Umeå, Sweden; ^2^Department of Epidemiology and Global Health, Umeå University, Umeå, Sweden; ^3^Department of Social Work, Umeå University, Umeå, Sweden; ^4^Centre for Demographic and Ageing Research, Umeå University, Umeå, Sweden

**Keywords:** police, education, mental health, law enforcement, police training

## Abstract

**Background:**

The stressful and complex nature of police work and its adverse effects on mental health are well-documented in police research. The mental health of police students however, has not been given the same attention. To the best of our knowledge, studies on the mental health of Swedish police recruits have not been undertaken since 2010.

**Objectives:**

The present study aims to examine whether there are differences in the mental health between two cohorts (2009 and 2020) of Swedish police recruits, as well as to compare the mental health of both cohorts with the general population data collected in 2002.

**Methods:**

Data was collected using the SCL-90-R survey. Data was analyzed using multivariate analysis of variance (MANOVA) and independent sample *T*-tests. Bi-variate analyses including *t*-test and chi-square were used to examine differences in sociodemographic variables between the two cohorts.

**Results:**

A total of 376 police recruits participated in the study. Results indicated no significant differences between the cohorts with regards to the three global indices of the SCL-90-R: Global Severity Index (GSI), Positive Symptom Total (PST), and Positive Symptom Distress Index (PSDI). Recruits with a college degree had lower scores on GSI and PSDI, similar to respondents that where in a relationship vs. singles. A total of 15 (four female) recruits had GSI scores above the Swedish patient mean. Compared with the general population, males and females from the 2009, as well as females from the 2020 cohorts had lower or insignificantly different mean scores on all global indices, with males from the 2020 cohort having a significantly lower PST score.

**Conclusions:**

While the vast majority of recruits had results that where indicative of a low prevalence and intensity with regards to mental health disorders, some recruits did score above the Swedish patient mean. While mental preparedness is part of the curriculum for Swedish police recruits, interventions targeting the stigmas of poor mental health could be of value. The fact that educational attainment appears to have a positive impact on the mental health of police recruits, could be taken in to consideration when recruiting future police officers.

## Introduction

The stressful and complex nature of police work is well-established in police research (1–7). Police officers are confronted with not only organizational stressors, such as lack of support from leadership, time pressure, and staff shortages, but also task-related strains, such as potential violence, threats, and exposure to unknown situations and interpersonal conflicts, which have been established since the infancy of police-related research (8–11). To handle the situations police officers are not only faced with but are required to manage, officers are given far-reaching mandates, including the lawful right to use force when conducting their duties. Given the power vested in the police, the discretion with which officers use this power is of great importance not only to the individuals with which they interact but also to public perceptions of trust in the justice system (12). With these mandates and the importance of discretion as a point of departure, the factors of conflict management and behavior play an important role. Notably, this is an area where officers have a breadth of possible approaches or behaviors ranging from the misuse of power to withdrawing from engaging in conflicts, and where a range of factors appear to affect the propensity of officers to use force (9, 13, 14). Notwithstanding the several investigations on different aspects of police work, less attention has been paid to the context of police education and how police officers are prepared for challenges they will meet in the line of duty. The lack of knowledge about police education might depend on variations in police education structures with regards to length and content in many countries, as well as the fact that police research is a relatively novel area of research.

## Background

### Basic Training for Police Officers

In Sweden, police officers go through 2 years (four terms) of formal training at one of the universities that have a basic training program for police officers. After graduation, future officers go through 6 months of probationary training, whereafter (if given a passing grade), they are hired as sworn officers.

Since 2006, and the inception of the *National Basic Tactics* [Nationell Bastaktik], all recruits and officers have received training in a mental preparedness program, which includes basic knowledge of stress and reactions to stress, coping, mental preparedness (short- and long-term), mental training through mental and muscular relaxation, and methods for improving sleep quality (15). The training to be a police officer is conducted at five universities and is regulated in broad terms by an education plan provided by the Swedish Police. Therefore, the material described above can be supplemented by other sources, as long as they are in line with the goals and contents of the plan (16) and the competency profile for police officers (17).

### Declines in Mental Health Status—From Recruitment to Officer

Corollary to the aforementioned demands and strains, as well as the power vested in them, aspiring officers generally go through rigorous testing. Although recruitment processes and methods of testing differ, commonly used test batteries include personality tests, such as the MMPI-2 (3) and the NEO-PI (18), physical fitness tests (19), and medical screening. Therefore, it is perhaps not surprising that, compared to the general population, police recruits at a group level are in good health upon admission (20, 21). However, research on the overall health of police officers shows that despite strong baseline results (i.e., upon admission to the academies), there appears to be a marked decline in the mental and physical health of police officers (1, 19, 22). Evidence has suggested that among the negative effects of police work are anxiety, depression, post-traumatic stress disorder (PTSD) (1, 7, 23, 24), burnout (10, 25), and substance abuse (26). It is well-known that mental health conditions, such as those described above, can be severely debilitating, significantly increase the risk of premature morbidity, and account for a large proportion of the burden of disease globally (27, 28). Mental health conditions have also been shown to have significant comorbidity with other non-communicable diseases ranging from (but not limited to) cardiovascular disease to oral health (29, 30). As already mentioned there appears to be less known about the mental health status of police recruits than about police officers. In reviewing previous research on mental health among police recruits a literature search was conducted in PubMed using the search terms ((mental health) OR (mental status) OR (stress) OR (psych^*^)) AND (Police/education[MeSH]). A similar search was conducted in ERIC and ASE using the search terms: (police education OR police recruit^*^ OR police trainee^*^ OR police student^*^) AND (mental health OR mental status OR psych^*^) with Subject terms: police, police education and higher education.

The absolute majority of the articles that were found examined various forms of training interventions for officers, and in some cases recruits, aimed at handling people suffering from poor mental health, or psychiatric disorders and *Crisis Interventions Teams* (CIT). Research in to the mental health of police recruits were however scarce. One study examined the association of mental status of police recruits and periodontal health (29) with other (*N* = 3) examining the associations of personality and mental health (20, 21, 31). In other studies coping behaviors of police recruits during training have been examined, suggesting that the training is a potentially stressful environment (32, 33), with recruits reporting an increase in alcohol consumption in part to cope with stress (33). However, little remains known about the mental health and symptoms of distress among police recruits.

### Mental Health and Police Officer Performance

The deleterious effects of mental health conditions mentioned above are however not limited to the individual, and in the case of police officers, these effects can be amplified by their work context. As already stated, conflicts and confrontations are omnipresent features of police work and need to be handled in a professional manner (34). Extant research on the mental health conditions described above shows that, for example, PTSD and burnout can have significant negative effects on conflict and conflict management behavior and performance (10, 35, 36). Finally, the deleterious effects of poor mental health on police officers could further be amplified by limited leeway in terms of feasible alternatives for coping strategies and help-seeking, where officers are: (1) required to face conflicts and other stressors daily (thus limiting the possibility of situational coping); (2) the stigmas surrounding mental health issues among police officers appear to be strong (1, 37, 38); and (3) police cultures where the potential dangers facing police officers are emphasized, adding an impetus for the development of a view of the world as a dangerous place where a “warrior” or “combat mindset” is necessary (39, 40) and where fearlessness and stoicism is encouraged (41).

Thus, the poor mental health of police officers and declines that have been shown in terms of mental health (42) and the negative effects that are perhaps particularly salient among police officers compared to the general public make police officer mental health a public health issue that should be paid great attention, especially in the recruitment process and training of police officers. Physical fitness plays an important role in resilience to stressors and positive effects on mental health (43) and performance, already in training (44), as well as good baseline values in terms of mental health. Even small negative changes in mental health could have negative effects on job performance and resilience among police officers.

### Relevance of the Study

The present study is part of an ongoing research project that started in 2018 with the aim of understanding the interplay between conflict management and mental health among Swedish police officers and recruits. To the best of our knowledge, the most recent research on the mental health of police recruits in Sweden (post-admission) was conducted in 2010. During this time, changes in society and recruitment have occurred that could affect the mental health of police recruits. For example, in Sweden, recent studies have shown that while overall rates of homicides in Sweden have decreased since the 1990s, gun-related homicides and attempts to kill have gone up among males, with indications that the increase in part is attributable to gang-related conflicts (45). According to official statistics, the rate of gun-related homicides has seen an approximately two-fold increase between 2011 and 2018, with Sweden having comparatively higher per capita rates of individuals killed due to gun violence than almost all other European countries (46, 47). Sweden has also seen an increase in the use of hand grenades and explosive devices (48). These changes could have an adverse effect on the perceived dangers of the task environments that officers are required to operate within, thus affecting the mental health of future officers.

In addition, in recent years, the Swedish Police Authority has faced difficulties recruiting new, potential police officers. For example, between 2019 and 2020, several positions at the schools were not filled. This raises the question of whether less competition for places at the academy could have had a negative effect on the health of those who have been admitted to the schools. Furthermore, the criteria for admission have undergone changes between the last study in 2010 and the present day, most notable among these changes are perhaps a lowered minimum age for admission from 20 to 18, removed language test (17, 49), and lowered minimum score on the UNIQ-test, which is intended to measure the *g-factor* (50); the minimum score for admission has been lowered from 4 to 3, thus requiring a score lower than the population mean (51).

In the present study, data from a cohort from 2009 were compared to data collected from another cohort in 2020. While the cross-sectional design precludes causal inferences between the changes now described and differences in symptoms of psychological distress among recruits, the aforementioned changes make comparisons between the cohorts relevant (52). Moreover, while it can be assumed that police recruits, given the recruitment process they have undergone, are likely to have a lower prevalence of mental health disorders, keeping in mind the responsibilities that come with the job and the challenges that officers face—simply assuming is not enough. Given that very little attention has been given to the mental health and distress among police recruits, there appears to be a gap in knowledge as to if the negative effects of police work are present already during training when students are faced with the potential dangers and risks with their future work.

### Research Aims and Questions

The overall aim of the present study is to examine if there are any differences in the psychological distress of police recruits in two cohorts, as well as in comparison with the general population, in order to deepen our understanding and knowledge of the importance of mental health and its potential negative consequences on police recruits. The control variables include age, gender, and educational attainment.

## Methods

### Data Collection

In the present study, two cohorts of police recruits, one from 2009 and one from 2020, will comprise the study sample; the duration and overall contents in terms of mental preparedness training were the same for both cohorts. Respondents in the 2009 cohort were recruited from one of three universities in Sweden that, at the time, provided basic training programs for police officers. Respondents in the 2020 cohort were recruited from four out of five universities that currently provide basic training programs for police officers. Participation was voluntary for both cohorts, and all respondents received information on the aim and scope of the respective studies. Respondents were also asked to fill out a written consent form before completing the included surveys. Both cohorts filled out sociodemographic questionnaires as well as the Symptom Checklist (SCL-90-R).

Data collection for the 2020 cohort was initiated in early 2020; initially, the data collection was intended to include more recruits as well as police officers in Sweden. However, the COVID-19 pandemic forced a halt to data collection through face-to-face meetings, and instead the surveys were transferred to an online survey platform: LimeSurvey. Recruits were then contacted through their respective schools and asked to participate in the study. Recruits were asked to complete the same survey, and written consent was obtained through the LimeSurvey platform. Data collection for the 2009 cohort was conducted during face-to-face meetings.

### Survey Measures

#### Sociodemographic Questionnaires

The sociodemographic questionnaires were developed independently and contained questions specific to each one. Both questionnaires included information on age (continuous), gender (“male,” “female”), marital status (“single,” “other,” and “married”), educational attainment (“high school” or “college”), and children (“parent” or “no children”), which could thereafter be compared between the two cohorts.

#### SCL-90

The SCL-90 was developed by Derogatis and Cleary (53) as an instrument to assess psychological problems and symptoms of psychopathology in both patient and general populations. The instrument consists of 90 items, with each item scored on a five-point Likert scale ranging from 0 = “not at all” to 4 “extremely.” In the present study, a Swedish translation of the instrument was used. The Swedish version of the instrument has been validated and psychometrically evaluated, and normative data from both patients (*N* = 1,782) and the general population (*n* = 1,016) are available (54). For the present study one justification for using the SCL-90 was the availability of previous data in a comparable cohort, thus enabling comparisons between both general population as well as a similar cohort (20). The SCL-90 measures nine dimensions of symptoms: somatization, obsessive-compulsive symptoms, interpersonal sensitivity, hostility, depression, anxiety, paranoid ideation, phobic anxiety, and psychoticism. Results from the SCL-90 can also be divided into three global indices that are indicative of psychopathology and level of distress: the Global Severity Index (GSI), Positive Symptom Index (PST), and Positive Symptom Distress Index (PSDI). The GSI reflects overall psychological distress, regardless of dimension, and Positive Symptom Total (PST) is the number of items answered positively (above 0). The PSDI indicates the intensity of symptoms, and it is calculated by dividing the total score by the number of positive items (54, 55). Previous studies on psychometric properties of the instrument have shown mixed results in terms of the factor structure of the nine subscales, limiting its use for more detailed discrimination between symptom groups. However, several studies have verified its usefulness as a screening tool, as well as for between-group comparisons in both clinical and research settings, with the global indices (GSI, PST, and PSDI) being particularly useful for assessing mental distress and the presence of symptoms of psychopathology (54, 56, 57).

#### Ethical Approval

Ethical approval for the present study was obtained from the Swedish Ethical Review Authority, with protocol number 2019-05208 for the 2020 cohort, and the Ethics Committee at Umea University approved the study protocol (Dnr 08-018M) for the 2009 cohort.

### Statistical Analysis

The data were analyzed using R (58) and STATA (59). We applied bivariate analyses, including *t*-tests and chi-square tests, to examine the differences in continuous and categorical sociodemographic variables, respectively, between the two cohorts (Table 1). Cohen's *d* was used to assess the magnitude of differences (60). We used the values originally stipulated by Cohen as a rough guideline in determining magnitude with the following levels of *d*: 0.2, 0.5, and 0.8 indicative of small, medium and large effects, respectively (60, 61). Statistical significance was defined as *p* < 0.05.

**Table 1:** Distribution of sociodemographic characteristics by cohort.

**Characteristic**	***N***	**Overall, *N* = 376**	**2020, *N* = 376**	**2009, *N* = 101**	***p*-Value**
		***N* (%)**	***N* (%)**	***N* (%)**	
**Gender**	376				0.09
Male		246 (65%)	180 (65%)	66 (65%)	
Female		130 (35%)	95 (35%)	35 (35%)	
**Degree**	376				<0.001
**High school**		256 (68%)	210 (76%)	46 (46%)	
College		120 (32%)	65 (24%)	55 (54%)	
**Marital status**	376				0.4
Single		164 (44%)	116 (42%)	48 (48%)	
Relationship		133 (35%)	103 (37%)	30 (30%)	
Other		79 (21%)	56 (20%)	23 (23%)	
**Parent**	376				0.044
No		315 (84%)	224 (81%)	91 (90%)	
Yes		61 (16%)	51 (19%)	10 (9.9%)	
		**(mean/±SD)**	**(mean/±SD)**	**(mean/±SD)**	
**Age**	376	27.1 (5.2)	27.9 (5.5)	25.1 (4.0)	<0.001

*P-values indicate differences between cohorts*.

Multivariate analysis of variance (MANOVA) was conducted with cohort, gender, parent, and educational attainment as independent variables and the three global indices (GSI, PST, and PSDI) as dependent variables to assess differences between cohorts and sociodemographic factors.

Prior to running the MANOVA, data were explored to ensure that the necessary assumptions for the test were met. To detect multivariate outliers, Mahalanobis distances were calculated (*p* = 0.001 and *DF* = 5). A number of outliers were found in both cohorts (*n* = 6 for 2020 and *n* = 3 for 2009) and were removed from the subsequent analysis. Data from both cohorts were also compared to general population data using gender-stratified one-sample *t*-tests, and data from the general population were collected from Fridell et al. (54).

## Results

The sample for the study consisted of two cohorts of police recruits (2009 and 2020) with a total of 376 police recruits from a Swedish basic training program for police officers (see Table 1). The original dataset contained 400 respondents in total, but 24 were removed because of missing sociodemographic variables.

### Mental Health Compared to General Population

Data from the present study were compared to general population data (from 2002) by gender and cohort in the corresponding age groups. In the 2009 cohort, both males and females had lower or insignificantly different mean scores than the general population. Males scored higher on obsessive compulsion than the general population (*t* = 2.4794, *p* = 0.0158, effect size = 0.31). As for the 2020 cohort, females similar to the 2009 cohort had lower or insignificantly different mean scores across the subscales and global indices, except for obsessive compulsion with borderline significant results (*t* = 1.9882, *p* = 0.05, effect size = 0.23). Their male counterparts had less homogenous differences, with significantly lower scores in interpersonal sensitivity, hostility, and PST, and significantly higher scores in psychoticism, anxiety, and depression (see Table 2). Thus, males from the 2020 cohort stood out when compared to the general population of the same gender, even though females in both cohorts as well as females in the general population overall had higher means in all sub-scales as well as global indices compared to their male counterparts.

**Table 2:** SCL90 means.

**Subscale**	**Male**					**Female**				
	**General pop**.	**2009**	***d***	**2020**	***d***	**General pop**.	**2009**	***d***	**2020**	***d***
Som	0.41	**0.28**	−0.40	**0.25**	−0.51	0.49	**0.27**	−1.05	**0.32**	−0.502
Sub2	0.48	**0.64**	0.31	0.54		0.57	0.58		**0.69**	0.225
Sub3	0.33	0.31		**0.41**	0.21	0.49	0.40		0.46	
Sub4	0.39	0.48		**0.46**	0.16	0.72	**0.56**	−0.36	0.64	
Sub5	0.33	0.34		**0.40**	0.20	0.56	**0.39**	−0.54	0.48	
Sub6	0.29	**0.10**	−1.25	**0.17**	−0.52	0.34	**0.08**	−1.61	**0.15**	−0.896
Sub7	0.09	**0.06**	−0.25	0.11		0.18	**0.09**	−0.68	0.17	
Sub8	0.32	**0.20**	−0.41	0.35		0.38	**0.17**	−0.66	0.32	
Sub9	0.12	0.16		**0.16**	0.16	0.18	0.16		0.19	
GSI	0.33	0.32		0.36		0.45	**0.33**	−0.48	0.44	
PST	23.4	**18.9**	−0.31	**20.7**	0.16	26.5	**19.9**	−0.56	**20.6**	−0.369
PSDI	1.38	1.41		1.33		1.43	1.42		1.42	

*General population data from Fridell et al. (54). Bold text indicates significant p-value (p < 0.05) for mean-differences between general population and respective cohort*.

### MANOVA

A one-way MANOVA was performed to assess differences in mental health between the cohorts. Box's M was used to check the homogeneity of covariance assumption, with a given *p*-value < 0.001, indicating that the assumption was violated. Therefore, Pillai's Trace was used, as it is robust to such violations (62). Pillai's Trace with only cohort as the fixed factor showed a significant difference between with only cohort as the fixed factor showed a significant difference between the two cohorts [*V* = 0.027, *F*_(3, 327)_ = 3.042, *p* = 0.029]. A test of between-subjects effects, however, showed that the differences for GSI and PSDI were insignificant (*p*= 0.242 and 0.343, respectively) with PSDI being borderline significant (*p* = 0.053). Subsequent MANOVAs using Pillai's Trace and the remaining sociodemographic variables, as fixed factors, showed significant differences in educational attainment [*V* = 0.046, *F*_(3, 321)_ = 6.627, *p* ≤ 0.001] and marital status [*V* = 0.046, *F*_(3, 322)_ = 1.329, *p* = 0.02]. The main effects were significant for GSI (*p* = 0.008) and PSDI (*p* ≤ 0.001), with respondents with a college degree having lower means than those with a high school degree. The mean PST did not show statistical difference between categories of educational attainment (*p* = 0.542) (Table 3). Regarding marital status, the main effect was similarly significant for GSI and PSDI (*p* = 0.007 for both) and insignificant for PST (*p* = 0.136). Being in a relationship (married or living together with a partner) or otherwise (in a partnership but not living together, unmarried) were associated with lower GSI and PSDI and insignificant differences for PST.

**Table 3:** MANOVA (Crude and adjusted means).

	**Indices**					
	**GSI**		**PSDI**		**PST**	
	**Crude mean**	**Adjusted mean**	**Crude mean**	**Adjusted mean**	**Crude mean**	**Adjusted mean**
**Cohort**						
2009	0.327	0.179	1.374	1.284	19.701	11.808
2020	0.368	0.205	1.338	1.263	23.081	14.066
**Gender**						
Male	0.348	0.289	1.332	1.314	21.892	18.469
Female	0.375	0.309	1.383	1.363	22.602	18.789
**Degree**		0.240				
High school	0.395	0.144	1.342	1.261	24.467	15.889
College	0.272^***^		1.362^***^	1.286	16.904	9.986
**Parent**						
No	0.374	0.229	1.356	1.295	23.011	14.933
Yes	0.253	0.155	1.301	1.252	16.667	10.942
**Marital status**						
Relationship	0.301^*^	0.162	1.341^*^	1.288	19.220	11.234
Single	0.425	0.261	1.380	1.313	25.564	16.308
Other	0.297^*^	0.153	1.288^*^	1.219	19.297	11.269

* *p < 0.05*,

*** *p < 0.001*.

### Scores Above Cut-Off for Swedish Patient Data

Using the mean scores of the Swedish normative data for the patient group as a cut-off, respondents with scores above the patient level for GSI (1.02 for males and 1.21 for females, respectively) were extracted from the data. A total of four females (all in the 2020 cohort) and 11 males (*n* = 3, 2009) were identified. Given the low numbers, statistical comparisons within the group were not deemed relevant, but interestingly, only one individual had a college degree, none had children, and one respondent was aged > 30. Scores among the respondents ranged between 1.1 and 1.8 for males and 1.2 and 1.6 for females.

## Discussion

In the present study, we aimed to examine the differences between two cohorts of Swedish police recruits, using control variables such as age, gender, children, and educational attainment. The study has some limitations that should be addressed when analyzing the results. The SCL-90-R measures psychological distress and symptoms of psychopathology at a very limited time (7 days). Thus, the results are at least on an individual level more sensitive to being affected by temporary fluctuations in psychological distress and therefore represents a snapshot of the psychological distress of the recruits. Also, the differences in the size of the samples could be a factor in the results, with smaller group sizes yielding less power in the analysis. However, given the lack of previous research on the topic, the results can be of importance not least for future research.

While the multivariate analysis between the two cohorts was significant, follow-up univariate analysis showed that only the difference in the number of positive symptoms (PST) was borderline significant at *p* = 0.053. This despite the fact that, at a societal level, there has been an increase in mental health disorders in the period of 2006–2020, in the comparable age group of 16–29 years old (63). These differences could be mitigated by the tests that the recruits go through before admission, despite the changes in recruitment and the increase in the number of recruits admitted.

Educational attainment appeared to have a positive effect on the psychological distress of police recruits, with significant differences for both the GSI and PST indices. While this study does not provide answers as to why educational attainment appears to be a protective factor in terms of prevalence of symptoms of mental health disorders, the results can still be of interest in terms of how to recruit police officers, and the value of higher education for police officer performance and longevity. This also adds to extant research on police officer performance in conflict situations, where educational attainment has been shown to have a positive effect on the use of force and verbal communication, where officers with a higher education degree are less likely to use coercive communication, use of force, and are less likely to receive complaints from the public (64, 65).

Compared with the general public normative data from 2002, recruits from both cohorts had mean GSI scores that were insignificantly different (lower for 2009 males and females, higher for 2020 males) from the Swedish general population, except for females in the 2009 cohort that had a significantly lower mean. Recruits from both cohorts, regardless of gender, also reported fewer psychopathological symptoms than their general population counterparts. This is perhaps not surprising, given that recruits have undergone rigorous testing, including psychological evaluations. The fact that the male 2020 cohort scored significantly higher on some subscales than their male counterparts from the general population is, however, something that could warrant further attention, even when taking the small effect sizes in to account. For example, with the exception of females in the 2009 cohort, the mean scores for obsessive compulsion were higher than the general population in the corresponding age group. This could for example be indicative of an educational climate that fosters fear of making mistakes and hampers the ability to take initiative and think freely. On a positive note, the low score across both cohorts, regardless of gender, with regard to hostility compared to the general population, could have several positive effects. Notably, previous studies have shown a relationship between anger/hostility and vulnerability to PTSD (36). A possible alternative explanation for this finding is that the subjects included in the present investigation are amidst their education and have not yet been faced with the challenges present in the line of duty. Future studies should examine how these scores change when police officers face the darker sides of police work and society.

While the number of respondents who scored above the cut-off for Swedish patient means, and therefore showed symptoms indicative of psychological distress, was low (*n* = 15), it was still noteworthy and could have implications not only for the respondents in question, but for those that they will possibly interact with in the future. As police officers, these individuals will be exposed to high levels of stress, shift-work, and other risk correlates of serious mental health issues, such as suicide, in which police officers and other emergency personnel have been shown to have increased risks (66).

The fact that there were more males than females with these high scores could perhaps be indicative of a lower propensity to actively seek help for these issues, which could increase the severity of the symptoms in the long term. This finding is in line with extant research on police help-seeking behaviors that have shown considerable stigmas surrounding mental health issues and a macho-culture associated with police work, where even officers with suicidal ideation rarely sought psychological help (37, 67, 68). It is vital that police education programs effectively identify individuals who already suffer from mental health disorders during their educational time, and that these are given proper attention and support. Previous research has shown that one reason for maladaptive help-seeking behaviors could be because officers are under constant (perceived) scrutiny, and that fear of, for example, being disarmed, could lead to individuals hiding symptoms of mental health disorders (69). If mental health-related issues are addressed in an open and effective manner, police work is also rich in protective factors, such as camaraderie and support (66). However, for these protective factors to be effective, the stigmas need to be removed, thereby hindering social withdrawal and covering up symptoms of mental health issues. To our knowledge, the help-seeking behaviors and attitudes toward mental health issues among recruits have not been explored to the same extent as among police officers, and the results of the present study warrant further investigation into these issues.

Prospective, longitudinal studies following recruits from admission and into their careers could provide important answers on how these individuals are supported, to what extent they are discovered during training, and what implications the presence of psychopathology during training has for their future careers.

## Conclusions

While the vast majority of recruits in the sample studied had results on the SCL-90-R that were indicative of low prevalence and intensity with regard to psychological distress, a number of recruits reported scores above the Swedish patient mean. While studies focusing on psychological distress among police recruits are rare, these results add to previous studies that identify the time in training as a potentially stressful environment even for the recruits (33). In addition, with the data available in the present study, mean scores appeared to be affected by educational attainment as well as the relationship status of the respondents. This finding could be useful for the recruitment of officers, where educational attainment has previously been shown to positively influence conflict behaviors, but could also positively affect the mental health of future officers. Further studies should be conducted with regard to the help-seeking behaviors of police recruits and the educational climate at the schools, where recruits appear to be under a lot of pressure, evident in high scores on obsessive compulsion, for example. While, at least in the Swedish context, mental preparedness is, in fact a part of the curriculum, interventions targeting the stigmas of poor mental health and the importance of seeking help could be of value. Such interventions, aimed at college students, have been shown to have positive effects both in the short- and long-term (70). While the studies on the topic of interventions aimed at resilience training for police recruits are scarce, previous studies have suggested that such interventions are likely most effective already early in the training of the recruits (71). While the declines in mental health of police officers are well-documented, our study - in line with the few previous studies that have been conducted on the topic, suggest that more research is needed and that this decline could start already during academy training.

## Data Availability Statement

The raw data supporting the conclusions of this article will be made available by the authors, without undue reservation.

## Ethics Statement

The studies involving human participants were reviewed and approved by Swedish Ethical Review Authority and the Ethics Committee at Umea University. The participants provided their written informed consent to participate in this study.

## Author Contributions

ME, MG, and MP conceived the study and where responsible for the first draft. MG, MP, and A-KH contributed by commenting on the manuscript and approved the final version. ME and MP conducted the data analysis. All authors contributed to the article and approved the submitted version.

## Funding

This study is part of a Ph.D. research project funded by Umeå School of Education, Umeå University, and the Unit for Police Education, Umeå University.

## Conflict of Interest

The authors declare that the research was conducted in the absence of any commercial or financial relationships that could be construed as a potential conflict of interest.

## Publisher's Note

All claims expressed in this article are solely those of the authors and do not necessarily represent those of their affiliated organizations, or those of the publisher, the editors and the reviewers. Any product that may be evaluated in this article, or claim that may be made by its manufacturer, is not guaranteed or endorsed by the publisher.
